# T790M突变的非小细胞肺癌研究现状与前景

**DOI:** 10.3779/j.issn.1009-3419.2017.03.09

**Published:** 2017-03-20

**Authors:** 芹 于, 达 姜, 颖 李

**Affiliations:** 050011 石家庄，河北医科大学第四医院肿瘤内科 The Forth Hospital of HeBei Medical University, Shijiazhuang 050011, China

**Keywords:** 肺肿瘤, T790M突变, 三代TKI, 耐药, Lung neoplasms, T790M mutation, Third generation TKI, Drug resistance

## Abstract

目前，针对表皮生长因子受体（epidermal growth factor receptor, *EGFR*）突变的非小细胞肺癌（non-small cell lung cancer, NSCLC）公认的一线治疗方案是以EGFR酪氨酸激酶抑制剂（tyrosine kinase inhibitors, TKIs）为主的靶向治疗，尽管一代、二代TKIs带来的靶向治疗可为患者带来更长的无进展生存（progression-free survival, PFS），及更好的耐受，但其远期治疗不可避免会出现耐药。其中，50%以上的获得性耐药与T790M突变有关，因此美国国立综合癌症网络（National Comprehensive Cancer Network, NCCN）推出的最新指南已经提出三代TKI（Osimertinib，奥西替尼）可用于一线TKI治疗进展同时检出T790M突变的患者。但就在三代TKI为我们带来令人鼓舞的可长达13个月的中位PFS及延续着后EGFR-TKIs治疗时代的同时，也面临着严峻的挑战，如怎样实现T790M的检测及动态监测、对已有三代TKI的研究进展、出现三代TKI耐药的机制及后续治疗等，本文将围绕以上各热点问题展开综述。

## Abstract (en)

Targeted therapy with epidermal growth factor receptor tyrosine kinase inhibitor (EGFR-TKIs) is regarded as the main accepted first-line treatment on *EGFR* mutation in non-small cell lung cancer (NSCLC). Although targeted therapy for the first and two generation of TKIs may lead to longer progression-free survival (PFS) and better tolerance for patients, the long-term treatment will inevitably lead to drug resistance. Among them, more than 50% of acquired resistance is associated with T790M mutation. The latest guidelines from the National Comprehensive Cancer Network (NCCN) have been proposed that the three generation of TKI (Osimertinib) can be used in first-line TKI therapy progress with detecting T790M mutations in patients. Encouraged by the treatment time of the median PFS up to 13 months and sequential EGFR-TKIs treatment which brought from the three generation of TKI, we also face serious challenges, such as how to achieve the detecting and the dynamic monitoring of T790M, the research progress, mechanism of drug resistance and subsequent treatment of the existing three generation TKI *etc*. This article will revolve around the above hot issues.

目前靶向治疗无疑是肺癌有效且毒性较低的治疗手段。随着众多靶点的发现，在过去的50多年里晚期肺癌的中位生存期不断改善，既往诸多的临床试验提示，一代的表皮生长因子受体酪氨酸激酶抑制剂（epidermal growth factor receptor tyrosine kinase inhibitors, EGFR-TKIs）吉非替尼、厄洛替尼、埃可替尼，二代的阿法替尼，对于*EGFR*基因敏感突变的晚期非小细胞肺癌（non-small cell lung cancer, NSCLC）患者，在无进展生存（progression-free survival, PFS）、有效率方面，与传统化疗相比均有明显延长，同时患者生存质量及耐受性较好。然而，通常在靶向治疗后，患者会不可避免的出现获得性耐药。其中，以20号外显子上的T790M突变最为常见，因此，针对这一突变涌现出的以Osimertinib为代表的第三代TKI进入大家的视线，由于其临床疗效及可耐受的毒副作用，食品药品监督管理局（Food and Drug Administration, FDA）加速审批，批准其可作为一代TKI药物耐药后治疗策略^[1]^。但同时在三代TKI治疗过程中，也面临着诸多挑战和疑问。

## 1 T790M突变的耐药机制

EGFR-TKI耐药机制主要包括：①出现耐药突变，如T790M突变；②旁路激活；③表型改变，如腺癌向小细胞肺癌转化；④下游信号通路激活。其中，50%以上出现一代TKI耐药是由于出现了T790M突变^[2]^，即EGFR 20号外显子第790位点上的苏氨酸被蛋氨酸所取代（T790M），从而增强了ATP与EGFR-TK结合域的亲和力，导致EGFR-TKI不能有效阻断信号通路而产生耐药。同时T790M突变具有选择性，他是在TKI治疗过程中保留下来的耐药克隆^[3]^。多项研究^[3, 4]^发现，使用EGFR-TKI治疗前，若存在敏感突变L858R突变或19外显子缺失的同时存在T790M突变，则患者的PFS劣于不含有T790M突变组患者，但优于无EGFR敏感突变的患者，故该研究认为原发T790M突变是EGFR-TKI疗效的不良预测因子。同时针对T790M突变的定量检测可更精确指导临床，Lee等^[4]^研究发现，治疗前T790M的突变丰度与EGFR-TKI疗效及预后相关。基于TKI耐药机制的复杂性，突显出了T790M临床检测的重要意义。

## 2 T790M突变的检测及其动态监测

### 2.1 针对T790M突变的检测方法

《2016年的晚期原发性肺癌诊治专家共识》建议有条件的患者在疾病进展时应再次进行肿瘤组织活检，并进行病理和相关的基因检测以明确耐药的性质^[5]^。目前，T790M检测方法主要包括：①扩增阻滞突变系统法（amplified refractory mutation system, ARMS）；②聚合酶链式反应（polymerase chain reaction, PCR）；③二代测序法（next-generation sequencing, NGS）。ARMS法只能检测已知突变，方便快捷、特异度高，在临床广泛应用；而NGS是近年发展起来的高通量深度测序技术群，可一次性对几百万条至十亿条DNA分子进行并行测序，但对稀有突变及结构变异不敏感且价格昂贵，广泛应用仍受到限制。在标本选择上可有：组织、恶性胸水及血液，临床中多因组织获取困难，更多选择液体活检，NCCN等多项指南已经推荐使用NGS进行液体检测^[6]^。Rieux等^[7]^在厄洛替尼耐药的患者胸水中检测出T790M突变，并指出：胸水可以在取不到组织的情况下对患者进行耐药检测；另一项来自日本的研究数据指出胸水在所有类型标本中（除去纯DNA标本），其阳性率最接近石蜡组织标本，且其检测失败率比石蜡组织标本低，因此检测恶性胸水的ctDNA可能是那些临床难以获得二次组织病理患者的一个新选择^[8]^。若无组织及胸水标本，则可进行血液ctDNA检测，需要注意的是在血浆检测与组织检测中间存在互补性，即：因血液ctDNA检测存在较高的假阴性率，所以在一、二代TKI治疗失败后且血浆T790M突变检测是阴性的患者推荐行组织T790M突变检测^[9]^；但总体液体检测具有较高的敏感性，因此可作为组织检测的补充。

### 2.2 动态监测T790M突变

上文已经提到T790M突变是一代TKI疗效的不良预测因子，会影响总生存期，这提示我们是否T790M突变频率的动态变化会预测肿瘤细胞负荷的变化？是否会预知转移的发生？这两者间有何种关联，又有怎样的数值关系？在体外研究中，Chmielecki等^[10]^研究了肿瘤细胞中T790M阳性克隆比例对TKI敏感性的影响，研究发现，当T790M突变阳性的细胞占所有肿瘤细胞的10%以上时，会加速肿瘤细胞的增生；当T790M突变阳性的细胞占所有肿瘤细胞的25%以上时，则会明显降低肿瘤细胞对TKI治疗的敏感性。

Oxnard等^[11]^研究指出，一代TKI治疗后失败的NSCLC患者若检测出T790M突变阳性患者的进展后生存期显著长于无T790M突变患者。因此，T790M突变既是EGFR-TKI耐药的原因之一，也因为其可选择三代TKI进行后续治疗而因此成为进展后生存的良好预后因子。同时，还发现连续检测*EGFR*敏感突变患者血浆中的T790M突变，可以发现血浆T790M在影像学疾病进展的16周前已经出现；国内，Zheng等^[12]^则发现在一代TKI治疗失败后导致的临床进展前平均2.2个月可检测出T790M，这与国外的16周相比有所滞后，这给我们临床的靶向治疗带来了一个新的考验，那就是是否血液中一旦检出T790M突变就开始给予对应的药物干预，还是待肿瘤发展到影像学上的进展才开始改变治疗策略。在对EGFR-TKI治疗过程中，动态监测T790M基因状态变化的众多研究中可以得出，T790M突变是在TKI长期治疗过程中保留下的基因改变，他在疾病进展前6个月到进展后4个月表达阳性率进行性升高，同时伴有EGFR敏感突变（19外显子缺失或21外显子L858R）阳性率的大幅增加。因此，在TKI药效作用下，可能双突变共存有利于肿瘤细胞生长^[4, 12]^。动态检测T790M突变可能预测未来的耐药，可以进一步探究T790M突变诱导的耐药机制，并指导进展期NSCLC患者的治疗，但其中细则问题需要更多研究来论证，为使NSCLC治疗更个体化、更精准化，也需要血液T790M突变检出率灵敏度、特异性的提高。

## 3 针对T790M突变的三代TKI

### 3.1 Osimertinib（奥西替尼，商品名：Tagasso）

Osimertinib是一种强效口服的EGFR-TKI，可抑制*EGFR*敏感突变和T790M耐药突变，他通过与EGFR激酶区ATP结合域的半胱氨酸-797残基不可逆共价结合以抑制EGFR的活性；因*EGFR*突变体细胞的EGFR激酶区蛋白构象发生了改变，更易与Osimertinib结合，因此三代TKI类药物对野生型细胞降低影响^[13]^；同时体外实验也证明，Osimertinib与T790M突变的EGFR亲和力是野生型的200倍^[14]^。

在Osimertinib用于二线治疗的研究中，基于AURA试验对Osimertinib安全性及有效率的验证下，AURA2试验观察了210例EGFR-TKI治疗后出现疾病进展的T790M突变阳性的NSCLC患者，每日1次口服Osimertinib 80 mg直到疾病再次进展，主要研究终点客观缓解率（objective response rate, ORR）可达70%，其中6例（3%）患者可达完全缓解，140例（67%）患者部分缓解，最常见的3级、4级不良事件是肺栓塞（3%）、QT间期延长（2%）；严重的不良事件中应警惕间质性肺病^[15]^。进一步扩大样本的AURA3试验，Osimertinib较含铂双药显示出明显优势，中位PFS可延长5.7个月（10.1个月 *vs* 4.4个月），ORR可达71%；在起初伴有脑转移的144例患者中，Osimertinib的中位PFS较含铂双药组提高4.3个月（8.5个月 *vs* 4.2个月），不良反应降低^[16]^。AURA3试验与之前的AURA、AURA2试验结果保持一致，奠定了Osimertinib成为T790M突变阳性的晚期或转移性NSCLC患者新的标准二线治疗方案的基础，再次为患者带来“精准治疗”的福音。

Osimertinib能用于*EGFR*敏感突变患者的一线治疗，那么与一代TKI吉非替尼或厄洛替尼在一线治疗时孰优孰劣呢？一项Ⅲ期临床试验FLAURA正在进行，最终结果还未报告^[17]^。目前只有Ⅰ期临床试验结果，显示Osimertinib有70%的客观反应率，中位PFS可达19个月，可是若将三代TKIs应用于一线治疗也同样存在一些问题，例如当三代TKI出现耐药后，我们后续治疗还能有哪些，提早的应用三代TKI是否会导致我们在后续治疗的选择上受到局限？而在一代TKI治疗失败后，部分患者却仍能选择Osimertinib治疗。

针对脑转移方面，第一、二代的EGFR-TKI穿透血脑屏障的能力有限，但在2016年美国临床肿瘤学会（American Society of Clinical Oncology, ASCO）上报道了一项Ⅰ期临床研究BLOOM试验，用Osimertinib治疗NSCLC患者脑膜转移，入组了局部晚期*EGFR*突变的NSCLC，且既往使用EGFR-TKI治疗后出现疾病进展、并通过脑脊液检查确定为脑膜转移的患者，给予每日1次口服Osimertinib 160 mg，现共入组21例患者，治疗后7例有影像学确诊的肿瘤缩小，同时5例患者观察到神经症状改善，2例患者连续2次的脑脊液检测肿瘤细胞均呈阴性，数据截止时（2016年3月10日），15例患者仍在治疗，其中7例治疗时间超过9个月；总体，Osimertinib临床疗效好，其穿越血脑屏障的能力强于第一、二代TKI，对BLOOM研究进一步分析发现，9例患者中有6例观察到脑脊液中EGFR突变水平降幅超过50%，其中5例观察到持续降低，这与既往Osimertinib穿越血脑屏障的临床前数据相符，证明了Osimertinib在伴有中枢神经系统转移的难治性患者中的前景。同时患者服用Osimertinib，普遍耐受好，常见副作用有皮疹、甲沟炎、眼睛干涩等^[18]^。但我们也必须警惕少数患者间质性肺炎的发生，已有个案报道口服Osimertinib治疗后出现间质性肺炎并引起严重的胸闷、气短症状，这需要及早发现、治疗，必要时减量^[19]^。Osimertinib为患者提供了用EGFR-TKI序贯治疗的机会，延缓了接受化疗和其他治疗的时间。

### 3.2 rociletinib（CO-1686）

另一个原本可与Osimertinib抗衡的明星产品rociletinib（CO-1686），是口服的、不可逆的、靶向共价抑制剂，能够抑制激活突变和T790耐药突变，使野生型EGFR信号闲置。Sequist等^[20]^在Ⅰ期TIGER-X试验中对CO-1686在治疗既往接受过抗EGFR治疗的晚期或复发性NSCLC患者的疗效、安全性等方面进行评估，其观察到ORR可达59%。但当该药物进入更大的Ⅱ期试验时，因为进入更大的中心，纳入了更多的患者，使得最初反应率60%这一数据没有达到，只有30%左右，较为严重的毒副作用是高血糖和心电图QT延长，这也为其尽快上市带来了阻碍。因此2016年4月，FDA肿瘤药物咨询委员会投票反对rociletinib在T790M阳性的NSCLC患者中的加速审批决议，所以rociletinib能否有其真正的临床价值，还是只是昙花一现，值得期待。

### 3.3 Avitinib（艾维替尼，AC0010）

Avitinib是我国自主研发的第三代EGFR-TKI靶向药，他能够克服T790M突变所致的耐药。在2016年欧洲临床肿瘤协会年会（European Society for Medical Oncology, ESMO）学术年会上张力教授也对该药的研究进展进行了口头报告，截止至2016年5月共入组51例*EGFR*突变阳性且对一代TKI耐药的NSCLC患者（均在治疗前使用组织活检分析T790M状态，给予50 mg *qd*到600 mg *qd*爬坡剂量，数据截止时，总体的ORR为38.2%，T790M突变阳性的患者ORR可达62%，疾病控制率75%，最长持续缓解时间大于11个月；常见的不良反应有腹泻、皮疹，且呈剂量依赖性；其中250 mg *bid*或300 mg *bid*给药方案疗效较好，被推荐为Ⅱ期研究的用药剂量。剂量扩大试验正在进行中^[21]^。因此，对于一代TKI耐药的T790M突变型NSCLC患者，Avitinib治疗是安全的，其抗肿瘤活性也值得期待。

## 4 导致三代TKI耐药的主要机制及后续治疗

针对T790M突变的NSCLC患者，Osimertinib的反应率可达61%，然而，在治疗10个月左右会再次出现对于三代TKI的耐药^[1]^，而研究获得性耐药机制是当下研究重点，可能发生的耐药主要包括耐药基因突变、旁路激活及组织学转变等。

### 4.1 C797S突变及后续治疗

Janne等^[1]^通过对患者ctDNA的检测发现有40%的患者耐药机制为合并有C797S突变，27%的患者体内已无法检测到单纯的*EGFR* T790M突变基因，而其余33%患者体内并未检测到C797S突变基因，这说明针对T790M突变发生耐药的机制具有复杂性。C797S是位于EGFR 20外显子的一个位点，这个突变能阻挡Osimertinib与EGFR的结合。Thress等^[22]^针对ARUA试验中使用Osimertinib治疗病情进展前后患者的cfDNA进行比对，发现存在C797S突变。随后多项研究也证实C797S突变是T790M突变NSCLC患者对OSIMERTINIB及其他三代TKI产生获得性耐药的主要机制，包括3种分子亚型：①获得性C797S突变以及仍然存在*EGFR*敏感突变和T790M突变；②继续存在T790M突变以及*EGFR*敏感突变，无获得性C797S突变；③仍然存在*EGFR*敏感突变，但T790M突变消失以及无获得性C797S突变。在同时存在有*EGFR*敏感突变、T790M突变和C797S突变的细胞系中，发现其对目前所有的EGFR受体拮抗剂都有耐药，但是部分对西妥昔单抗敏感，但是西妥昔单抗或是以西妥昔单抗为主的综合治疗的了疗效还需进一步试验结果来支持。一个新的细胞系：MGH121，起初对三代TKIs（Osimertinib、CO-1686等）均敏感，随着TKIs加量，MGH12细胞出现耐药，当L858R/T790M/C797S同时存在突变时，该细胞系对所有EGFR-TKIs耐药；分开进一步研究，发现在体外系统，del19/T790M的细胞对第一、二代TKIs耐药，而del19/C797S则抵抗第三代TKIs^[23]^。因此，对肿瘤细胞基因的突变状态的分析可指导临床治疗。

随着耐药基因C797S的发现，第四代的TKI也孕育而生。EAI045，他是一个变构抑制剂而不是ATP竞争性的拮抗剂，主要作用于L858R/T790M突变型肿瘤细胞，与EGFR野生型相比，突变型与EAI045的结合力增强近100倍^[24]^。在一个含有L858R/T790M/C797S突变的小鼠模型中^[25]^，EAI045与西妥昔单抗联合应用，可使肿瘤明显缩小，有效率高达80%。这也与上面所提及的西妥昔单抗可对部分T790M/C797S突变以及同时存在*EGFR*敏感突变的细胞发挥作用相一致，为克服目前无敏感性的TKIs带来曙光。

### 4.2 其他三代TKI耐药的机制及后续治疗

因为对于三代TKIs耐药病例的不断积累，越来越多的耐药机制进入人们眼帘，除了常见的C797S突变以外，有研究证实C-Met扩增、HER-2扩增、*EGFR* L718Q突变可能均与三代TKIs获得性耐药相关^[26, 27]^。现有关Osimertinib联合MEK抑制剂（selumetinib）对比单药Osimertinib治疗*EGFR*突变的进展期NSCLC患者的多中心开放性临床研究（NCT02143466）正在进行中，预计在2017年会有结果。

伴随对耐药后机制的不断发现与研究，可能越来越多的像EAI045这样的四代TKI会进入人类的临床试验，但是在出现耐药后，尽可能完善二次活检或是血液cfDNA的基因检测，以求寻找精准治疗的机会也具有重要意义。在现有条件下，出现三代TKI耐药后应该全面评估病情及患者耐受，如果可以可选择化疗；而在AURA临床研究中^[1]^，观察到T790M野生型患者使用三代TKI有24%的有效率，65%的疾病控制率，优于目前标准的二线化疗，所有一、二代TKI耐药之后直接选三代TKI也不失为一种选择。另外，为延缓TKI耐药，现已有一代TKI联合贝伐珠单抗的诸多试验，其中BELIEF试验是有关厄洛替尼联合贝伐珠单抗治疗有活性*EGFR*突变，伴和不伴有T790M突变的晚期NSCLC患者的一项Ⅱ期研究，主要研究终点是PFS，共入组109例患者，最终结论是T790M突变的患者中位PFS可达16个月，较无T790M突变的患者10.5个月明显延长，ORR为70.3% *vs* 79.2%^[28]^，因此T790M突变的NSCLC患者是否对于贝伐珠单抗更为敏感？亦或贝伐珠单抗的加入是否可增加肿瘤细胞对TKI敏感性？由此我们是否可以在三代TKI治疗T790M突变的NSCLC患者的早期即加入血管靶向药从而延缓TKI耐药？这些需要进一步试验论证。同时基于基因变异的情况，TKIs联合其他药物，如上文提及的三代TKI联合西妥昔单抗、MET抑制剂的相关临床试验均在进行中，这都可能会有临床获益。在2017版NCCN指南中已经指出TKI治疗失败的患者可以选择阿法替尼+西妥昔单抗作为后续治疗^[29]^。

自从免疫治疗药物PD-1抑制剂获FDA批准用于NSCLC患者后，越来越的临床试验关注于TKI与免疫治疗药物联合应用的疗效。Tang等^[30]^及Lin等^[31]^研究发现*EGFR*突变组应用TKIs治疗，PD-L1高表达者比低表达者在ORR、疾病控制率、DFS、总生存方面更具优势，这提示了TKIs与PD-1/PD-L1免疫治疗结合的可能性。目前，多项PD检查点抑制剂与Osimertinib联合治疗的临床试验也处于进行当中，但现已发现这一联合的毒副反应发生率远高于预期，因此，这一联合具体的疗效及安全性还有待证实。在2016年ASCO大会上另一个很有前景的组合疗法是两种免疫治疗药物PD-1抑制剂（nivolumab）以及CTLA-4检查点抑制剂（ipilimumab），该试验的主要研究终点是安全性，次要终点是ORR及24周PFS，结果提示两药联合不良反应轻微，无患者出现治疗相关性死亡，且两者对于*EGFR*阳性突变的患者也显示出相应的治疗活性。因此nivolumab与ipilimumab作为进展期NSCLC的一线治疗方案是安全有效的^[32]^。所以这给我们一个启示，三代TKIs耐药后，也许在TKI基础上联合免疫治疗会是一个不错的选择。

## 5 总结

肺癌的发生、发展是一个多方向、多步骤的复杂网络系统，随着一代、二代、三代TKIs相继进入临床，患者的PFS、总生存及耐受均有明显改善，但接踵而来的问题就是患者对于靶向治疗的耐药。一、二代TKI治疗失败之后，发现了50%左右是由于T790M突变所导致的耐药，为针对这种获得性耐药机制，三代TKI以迫切的临床需求、良好的临床表现加快审批进入临床；那么接下来不可避免的就是对于三代TKI的耐药，现已经对其耐药机制有广泛研究，针对性的药物也已进入试验阶段，同时耐药后治疗方式的选择也都还在摸索着。总而言之，肺癌的靶向道路是在跌宕起伏中抱有希望，他需要我们充分了解EGFR-TKIs的耐药机制，综合应用靶向、化疗、放疗及免疫等多种治疗方式，寻找最佳结合模式，延长患者生存。
